# O037: Prevention of Staphylococcus aureus infection in NICU: routine microbiological surveillance and decolonization

**DOI:** 10.1186/2047-2994-2-S1-O37

**Published:** 2013-06-20

**Authors:** R Richtmann, SR Baltieri, CDA Silva, FB Mello, TT Rodrigues

**Affiliations:** 1 Hospital e Maternidade Santa Joana, Sao Paulo, Brazil

## Introduction

*Staphylococcus aureus* colonization is a risk factor for endogenous staphylococcal infection in vulnerable neonates. Several studies describe prophylactic measures for adult population, but very few recommendations are establish for neonatal intensive care unit (NICU). After detecting an increase in severe *S. aureus* infection in the NICU, the present study has the objective to check if a bundle of measures to decrease *S aureus c*olonization in NICU babies have impact in reducing infection.

## Methods

Prospective cohort from May/2011 to April/2012 (Period 2- P2) in a 70 beds Brazilian NICU. Weekly nasal swab to detect *S.aureus* colonization in all newborn admitted to the NICU and under intravascular catheter (peripheral or central) e/or mechanical ventilation. If positive culture, they were submitted to decolonization with nasal mupirocin ointment topical and oral hygiene with chlorhexidine 0.12% solution for 7 days. A nasal swab investigation after decolonization was performed as treatment control and if it persists positive a second decolonization treatment was indicated. Contact and droplet precautions were used to the methicillin resistant *S.aureus* (MRSA) colonized neonates. The *S.aureus* infection rate was compared to the previous year (Period 1-P1: from April 2010 to April 2011).

## Results

Both neonatal population in different periods presented similar device utilization ratio. On P1, 820 neonates were included, 28 (3.4%) presented *S.aureus* infection, 17 (2.0%) by MRSA and 11(1.3%) by methicillin sensitive (MSSA). On P2 (after bundle implementation), 1012 neonates were analyzed, *S.aureus* were diagnosed in 14(1,3%) , 5 (0.4%) MRSA (p=0.03) and 9(0.8%) MSSA (p=0.37), in comparison to P1. The colonization rate on P2 was 3.4% for MSSA and 5.3% for MRSA. There was no increase of infection related to other microorganisms. There was no adverse event related to the decolonization procedures.

## Conclusion

The *S.aureus* bundle including decolonization was effective in decreasing infection in the NICU babies, especially for MRSA infection. The impact on MSSA infection was lower. There was no microorganism replacement phenomenon.

## Disclosure of interest

None declared.

